# Supplementary material for: Are carbon emissions trading and green financial instruments synergistic? -Comprehensive quantitative research based on content analysis
Source: PLoS One. 2024 Mar 7;19(3):e0298601. doi: 10.1371/journal.pone.0298601 (PMC10919601; doi:10.1371/journal.pone.0298601)
Supplement: S1 Data — (ZIP) [file pone.0298601.s001.zip › Data/地级市Z_F工作报告.zip!/╣π╢1⁄2/│▒╓▌╩╨/└┤╫╘╣1⁄2╓┌║┼í╛┬φ┐╦╩2╛▌═°í┐_2009.docx]

各位代表：

现在，我代表市人民政府，向大会作工作报告，请予审议。并请各位政协委员和其他列席人员提出意见。

2008年工作回顾

2008年，是我市经济社会经受挑战和考验，继续保持平稳较快发展的一年。在省委、省政府和市委的正确领导下，在市人大、市政协的监督支持下，我们紧紧依靠和带领全市人民，认真贯彻党的十七大、十七届三中全会和省委十届二次、三次全会,以及市委十二届四次、五次全会精神，以解放思想学习讨论活动为动力，全面落实科学发展观，及时研究解决发展中的突出问题和困难，有效地促进经济社会平稳较快发展，圆满完成了市第十三届人大三次会议确定的主要目标任务。

一、经济综合实力稳步提高

经济平稳较快增长。全市实现生产总值442.8亿元，比上年增长12%，比全省平均水平高出1.9个百分点，增幅在全省排名由上年的第14位上升为第9位。人均生产总值1.73万元，增长11.2%。人民生活进一步改善，市区居民人均可支配收入11320元，增长8.9%；农民人均纯收入4977元，增长8.2%。

财税金融运行良好。地方财政一般预算收入16.02亿元，增长18.66%。各项税收总额42.38亿元，增长11.5%。其中，国税国内收入28.32亿元，增长10.37%；地税收入14.07亿元，增长13.85%。地方财政一般预算支出37.41亿元，增长11.52%。其中，公共财政支出27.65亿元，增长16.35%。年末，金融机构本外币各项存款余额476.8亿元、贷款余额160.1亿元，分别增长21.1%和12.7%。

县域经济持续发展。潮安、饶平、湘桥、枫溪分别实现生产总值189.4亿元、102.6亿元、38.8亿元和57.6亿元，增长13.5%、12%、8%和12%；地方财政一般预算收入分别完成4.44亿元、2.06亿元、1.45亿元和1.66亿元，增长29%、20.1%、18.1%和13%。潮安县被认定为全国食品工业强县、省旅游强县、省林业生态县，凤凰镇被授予“中国名茶之乡”称号。饶平县水族器材、玻璃陶瓷等产业发展势头良好，经济发展后劲逐步增强。

二、农业农村工作稳定发展

农业发展水平不断提高。全市粮食种植面积65.3万亩，总产24.7万吨，均超过省下达计划。茶叶、潮州柑、水果、花卉、畜牧等特色农业生产规模逐步扩大，种养结构不断优化。凤凰单丛茶地理标志申报通过国家评审并予以公示。农业技术推广、标准化建设和农产品质量安全工作加强。新认定无公害产品、绿色食品、有机食品共23项，累计131项。兽医管理体制、种子管理体制和基层农技推广体系3项改革取得新进展。渔业生产保持发展，深水抗风浪网箱养殖成效明显，水产品总量16.9万吨，增长5%。

农民收入持续增加。农业龙头企业、农产品流通企业带动能力增强，28家市级以上农业龙头企业带动农户7.1万户，户均增收1350元。现代农业示范园区建设加快，15个市级农业生产示范基地带动农户2.13万户，户均增收1957元。劳务经济加快发展，扶贫工作继续推进。

农村生产生活条件进一步改善。土地承包关系进一步完善，基层两委换届选举顺利完成。“三大涝区”整治工程即将陆续开工，一批防灾减灾工程得到实施。农村饮水安全工程建设稳步推进。镇通村公路硬底化建设全面完成，提前一年实现省的计划目标。行政村通班车覆盖率达到85%。新型农村合作医疗参合率达到98%。村庄规划进展顺利，农村环境逐步改善。

三、工业发展水平有新提升

规模经济逐步壮大。陶瓷、印刷包装、不锈钢、电子、食品、水族器材等特色产业保持发展，工业规模化、适度重型化程度提高，一批临港产业项目建设加紧推进。全市工业增加值239.2亿元，增长12.4%。其中，规模以上工业增加值131.3亿元，增长14.5%，增速居全省第10位。新增规模以上工业企业128家，累计1012家；新增产值超亿元企业16家，累计91家。规模以上重工业增加值52.5亿元，增长17.4%，增幅比轻工业高出4.8个百分点。

创新能力不断增强。技术改造步伐加快，年度技术创新改造项目98个，完成投资13.5亿元，增长28.4%。科技创新体系进一步完善，新增省级工程研发中心、企业技术中心、专业镇各1个，累计拥有量分别居全省第4位、第6位和第5位。省市共建陶瓷先进制造业基地已经启动。潮州陶瓷研究院完成部分建设并开展服务。新评定省级工艺美术大师29名。科技计划项目成果显著，获得省级以上科技项目立项79项，全省28个重大科技专项我市占有2项。信息化建设和专利工作继续加强，获得省现代信息服务业发展专项资金扶持项目2个，专利申请量、授权量分别居于全省第9位和第10位。

品牌战略有效实施。新增省级以上名牌名标54项，累计247项。新增“省产业集群升级示范区”2个。8项国家标准制修订获得立项，全国标准技术委员会水族工作组在我市设立。省食品、不锈钢器皿和厨具2个质量监督检测站建成并投入使用。

园区建设扎实推进。潮州市径南产业转移园、潮州港经济区总体规划完成初步成果。深圳（潮州）产业转移园整合工作基本完成。径南产业转移园首期开发建设工作准备就绪，天然气供应站及部分管网在园区建成。县、区工业园区规划建设继续推进。

四、第三产业逐步发展壮大

文化旅游成效明显。古城文化旅游区建设加快，牌坊街主体工程基本修复，许驸马府对外开放，市工艺美术研究所、潮州戏苑等纳入古城文化旅游线路。“广济桥”和“牌坊街”两大品牌逐步打响，潮州文化旅游核心竞争力进一步提高。旅游业发展态势良好，区域合作不断扩大，东山湖温泉度假村成为我市首家4A级旅游景区。全年旅游总收入35.1亿元，增长13%。

对外贸易克难前进。全市外贸进出口22.6亿美元，增长2.7%。其中，出口17.8亿美元，增长0.6%。出口结构调整优化，机电产品成为第二大类出口商品。潮州港被列为对台直航港口，大唐电厂5万吨级专用码头通过对外开放验收，通关环境进一步优化。

招商引资有新发展。新批外商投资项目24个，合同利用外资1.4亿美元、增长2.4%。增资扩产项目15个，合同增资额1.2亿美元。实际利用外资9187万美元，增长11.6%。与省属国有大型企业合作正在开展，在供水工程、污水处理设施、仓储物流中心建设和旅游业发展等方面达成协议。

市场消费保持旺盛。国内市场开拓取得新成效，规模以上工业内销产值368.3亿元，增长38.9%。汽车、商品房等大宗商品消费大幅度增长。全社会消费品零售总额170.6亿元，增长20.6%。物价涨幅得到控制，居民消费价格水平呈现前高后低走势，全年上涨5.4%。

五、可持续发展能力进一步增强

投资力度继续加大。全社会固定资产投资135.7亿元，增长12.1%。其中：工业投资76.2亿元，增长29.5%；更新改造投资62亿元，增长70.5%。重点项目建设扎实推进，41个年度重点项目完成投资56.1亿元，占年度投资计划的118%。500千伏韩江输变电工程提前动工，大唐电厂3、4号机组可望在今年上半年建成投产；厦深铁路、汕梅高速公路潮州段开工建设；韩江南北堤南堤段达标加固等水利工程建设进展顺利。设立市重大项目库，储备项目185个、投资总规模1271亿元，为增强发展后劲打下坚实基础。

环保节能成效明显。韩江、枫江、黄冈河流域污染综合整治稳步推进，污染源普查任务全面完成。关闭16家污染严重企业。节能节材、资源综合利用、陶瓷清洁生产和节能技术改造等工作扎实推进。单位生产总值和工业增加值能耗、二氧化硫及化学需氧量排放总量持续下降。生态环境逐步改善，城市环境定量考核成绩名列全省第3位，连续8年在省环保责任制考评中获得优秀等次。国家环保模范城市创建工作正式启动。

城市发展水平不断提高。《潮州市城市总体规划》等一批规划抓紧修编，城乡规划工作逐步加强，各类规划较好实施。绿榕南路、市开发区工业大道等城市道路建设积极推进，市垃圾卫生填埋场第二填埋区建设基本完成。宜居宜业宜游城市建设加快，创建“国家园林城市”和“中国人居环境范例奖”成果得到巩固发展。城市管理工作继续加强，奎元住宅区周边等城乡结合部环境得到有效整治，脏乱差现象有所改观，道路交通秩序和出租车营运市场进一步规范。

土地利用管理得到加强。局部调整土地利用总体规划。向省上报并获得批准用地261公顷，数量为近年来最多。强力查处整治违法违规用地和非法开采矿产资源，拆除违法违规建（构）筑物72.9万平方米，涉及用地面积1019亩，复耕复绿面积7740亩，违法违规用地蔓延势头得到有效遏制。

低生育水平保持稳定。落实层级动态管理责任制，完善计生利益导向机制，人口计生工作水平不断提高，潮安县、饶平县转类升级成果得到巩固。全市人口出生率10.98‰，自然增长率5.33‰，均控制在省下达指标之内。

六、和谐社会建设扎实推进

精神文明建设继续加强。认真开展解放思想学习讨论活动，深入学习实践科学发展观。大力弘扬新时期潮州人精神，评选出辉煌30年——潮州十大时代人物。韩江论坛和第二届“书香潮州”全民读书节成效明显。未成年人思想道德教育得到加强。文明创建活动广泛开展，一批单位受到上级表彰。

社会保障水平明显提高。各项社会保险费收入8亿元，增长13.7%。参加养老、失业、工伤、医疗保险人数分别增长9%、9.8%、10.3%和45.4%。城镇居民基本医疗保险成效显著,全市有25.2万人参保。被征地农民养老保险制度开始实施，有4973名群众纳入保障范围。最低生活保障人数增加、待遇提高，低保对象5.25万人，全年发放生活保障金5200多万元。经济适用房和廉租房建设加快推进，住房租赁补贴惠及市区550户“双困家庭”。送医、助学等慈善工作深入开展，扶危济困等爱心活动跃然成风。残疾人技能培训和就业工作加强，安置稳定就业人数832名。支援四川抗震救灾工作积极开展，捐款和援建资金超过7000万元。城乡统筹就业扎实有效，新增就业岗位2.12万个，下岗失业人员再就业1.11万人。城镇登记失业率2.49%，低于全省平均水平。农村劳动力培训转移步伐加快，培训2.03万人，转移就业4.88万人。《劳动合同法》较好实施，劳动合同签订率达到96%。

社会大局和谐稳定。始终保持高压态势，“两抢一盗”等严重违法犯罪活动受到有效打击。公安“三基”工程建设全面达标，社会治安防控能力进一步提高。各类社会矛盾较好化解，群体性事件得到妥善处置。“五小”专项整治扎实推进，药品放心工程积极实施，产品质量和食品安全工作不断加强。农信社改革继续深化，市华侨信托投资公司平稳撤销退市。安全生产形势稳定，应急管理工作扎实推进。

社会事业全面发展。城乡免费义务教育得到落实，受惠学生38.6万人。高中阶段教育普及率60.9%，居粤东各市首位。新增国家级示范性普通高中4所，新建扩建普通高中学校9所、职业技术学校5所，市技工学校被评为国家重点技工学校。文化工作取得新成效，农村和社区文化活动活跃，市文化艺术馆、博物馆主体工程基本完成。潮剧《曹营恋歌》在第十届广东省艺术节上获得8个奖项。枫溪区（瓷塑）、湘桥区意溪镇（木雕）被文化部命名为“中国民间文化艺术之乡”。新增省级文物保护单位2处、国家非物质文化遗产5项。公共卫生服务体系逐步健全，社区卫生服务不断发展，农村卫生站实现全覆盖。成功举办潮州星和陈伟南星命名、潮汕四市龙舟邀请赛等系列活动。双拥工作扎实有效，连续6届被省授予“双拥模范城市”称号。

依法行政继续强化。自觉接受市人大及其常委会的监督和市政协的民主监督。办理人大代表建议、政协委员提案等159件，满意率和基本满意率达100%。坚持依法行政，建立政府法律顾问等制度，制订实施规范性文件18件。完善市政府公众网功能，推进政务公开。建立行政审批电子监察系统，优化审批服务。实行政府投资非经营性建设项目代建制，提高项目投资效益。加强政府廉政建设，进一步完善惩治和预防腐败体系。

与此同时，人事编制、审计、司法、外事侨务、统计、物价、打私、广播电视、人防、气象、防震、地方志、档案、对台、民兵预备役、老龄、民族宗教、妇女儿童等工作有新发展。

各位代表，过去一年，我市发展遇到的困难前所未有，取得的成绩好于预期。这是省委、省政府和市委正确领导的结果，是全市人民同舟共济、共克时艰、团结奋斗的结果。这些成绩的取得，离不开市人大及其常委会和市政协的监督支持，离不开驻潮部队，以及各民主党派、人民团体和港澳台同胞、海外侨胞的鼎力支持。在这里，我代表市人民政府表示衷心的感谢并致以崇高的敬意！

我们清醒看到，经济社会发展中也存在一些突出问题和困难。一是经济总量不够大，产业结构不够优，抗风险能力不够强。二是受国际金融危机影响，外贸出口增长乏力，企业生产经营困难。三是节能减排任务仍然艰巨，违法违规用地、非法开采矿产资源等问题仍较突出。四是城市管理水平有待提高，农村生产生活环境亟待改善，社会治安管理仍需加强。五是财政减收增支因素较多，保民生、促发展压力大。六是一些部门超前意识不够，工作落实不力，办事效率不高。对于这些问题，市政府将进一步采取措施，认真加以解决。

2009年工作安排

2009年，是新中国成立60周年，也是实现“十一五”规划目标的重要一年。当前，国际金融危机的影响还在加深，这对经济外向度较高的我市造成极大冲击，我们的发展面临许多困难和压力。但是，金融危机带来的国际产业分工调整和企业并购重组，有利于我们以开放促发展，也为开拓国际市场提供新的机遇。中央实施积极的财政政策和适度宽松的货币政策，尤其是国家和省出台的扩大内需若干政策措施，对促进经济发展产生重大影响。出口退税政策调整和增值税转型改革，以及资源型和基础性产品价格的回落，对缓解企业经营困难、推动产业升级有积极作用。我们一定要坚定信心、迎难而上，在应对挑战中把握发展新机遇，在克服困难中赢得发展新优势，全力推动经济平稳较快增长。

今年政府工作的指导思想是：以邓小平理论和“三个代表”重要思想为指导，全面贯彻落实党的十七大、十七届三中全会和省委十届四次全会、市委十二届六次全会精神，深入贯彻落实科学发展观，以解放思想为引领，以改革开放为动力，按照“强信心、抢机遇，抓发展、保增长，重民生、促和谐”的要求，紧紧把握好发展的方向性问题和关键性工作，努力提升产业发展水平，不断增强可持续发展能力，切实加强管理服务，进一步改善民生，全力促进经济社会又好又快发展。

2009年，我市经济社会发展的主要预期目标是：生产总值增长10.5%，人均生产总值增长9.6%，单位生产总值能耗下降3.43%，地方财政一般预算收入增长13%，全社会固定资产投资增长16%，外贸出口总额与去年持平，全社会消费品零售总额增长20%，居民消费价格涨幅控制在4%以内，人口自然增长率控制在6.2‰以下，城镇登记失业率控制在3.5%以下，市区居民人均可支配收入增长8%，农村居民人均纯收入增长7%。

为实现上述目标，要扎扎实实抓好以下工作：

一、推进产业调整优化，提升经济发展水平

产业是经济的基础，要根据当前国内外经济形势和我市产业发展实际，全力加强产业建设。

（一）坚持改革发展，积极发展现代农业

以改革激发农村发展活力。稳定和完善农村基本经营制度，加快推进农村土地流转、集体林权制度改革。加强农村“一事一议”监督管理，探索建立“村企合作共建新农村”模式。落实惠农强农政策措施，加大“三农”投入力度，加强地质灾害防治、病险水库除险加固、机电排灌设施维护，加快农村生活垃圾、污水处理等基础设施建设，不断改善农村生产生活环境。

以产业化推动农业发展。保护和建设好基本农田，实施现代标准农田建设，改善耕地生产条件，稳定粮食生产。大力推进茶叶、水果、水产、畜牧、花卉等特色农业集聚发展，促进凤凰单丛茶、白叶单丛茶和潮州柑等特色产品的规模化、标准化生产。实施科技兴农战略，加强农产品质量认证和品牌建设，抓好动植物疫病防控工作。发挥农业龙头企业、流通组织的作用，带动农村经济加快发展。

以开发做大海洋渔业经济。加强现代标准化鱼（虾）塘建设，提升渔业发展水平。加快传统网箱改造，大力发展深水抗风浪网箱养殖。改造传统捕捞业，发展远洋渔业生产。加强海洋综合管理，完成海洋功能区划编制，完善海洋与渔业基础设施。

（二）加强服务创新，不断提高工业发展能力

加大服务扶持力度。继续引导企业增强战胜困难的信心，鼓励支持企业实施技术创新、完善内部管理、加强人才引进，在应对危机中转型升级。做好财税扶持、政策服务等帮扶解困工作，设立科技创新引导资金、节能专项资金，帮助企业发展、促进产业升级，重点支持50家规模较大、前景较好的企业提升发展水平。继续推进银企合作，引导金融机构加大信贷投放力度，积极帮助中小企业融通资金；采取市场运作、财政支持的办法，鼓励设立信用担保机构，努力解决中小企业融资担保难问题。

完善自主创新体系。推进国家陶瓷及水暖卫浴、包装产品等质量监督检测中心建设，发挥潮州陶瓷研究院和专业镇等公共服务平台作用，完善科技中介服务体系，为企业自主创新提供支撑。深化产学研合作，推进省市共建先进陶瓷制造业基地建设，支持发展一批技术改造和技术创新项目。加强服务引导，推动产业集群发展，提高产业集群的发展水平。

增强企业创新能力。落实企业研发费用税前抵扣等优惠政策，引导企业增加研发投入。鼓励企业设立工程中心、技术中心等研发机构，重视自主创新成果应用和产业化工作。支持企业实施各类科技项目、发展节能和资源综合利用项目。积极实施年度结转及新开工的88项重点技改创新项目，选择扶持一批陶瓷骨干企业实施工艺流程自动化改造，努力促进传统特色产业优化升级。

实施品牌标准战略。抓好区域品牌建设，力争新增2个省级产业集群升级示范区和2个省级火炬计划特色产业基地。鼓励企业争创名牌、经营品牌，支持有条件的企业打造国际品牌，实现品牌效益最大化。争取凤凰单丛茶地理标志保护获得国家确认，做好潮绣、潮州木雕地理标志保护申报工作。支持企业参与各类标准制修订，积极推行标准化生产。抓好产品质量和食品药品安全工作，严厉打击制售假冒伪劣商品行为。

加快推进产业转移。围绕《珠江三角洲地区改革发展规划纲要》的实施，研究制订对接的办法和措施，主动接受珠江三角洲地区辐射，积极承接发达地区产业转移。继续做好深圳（潮州）产业转移工业园整合工作，争取园区成为省级示范性产业转移园。加强各类产业园区基础设施和主导产业建设，争取中国房地产集团公司尽快投资开发径南产业转移园。加快各县区工业园的规划建设，努力解决中小企业发展用地难问题。

（三）加快建设和招商，努力形成临港产业支撑

坚持规划引导。加快完成潮州港经济区总体规划修编，以及中心区和重化工业园等主要功能区控制性详细规划编制，促进经济区科学有序建设。按照港口总体规划，加强港口岸线管理，提高岸线使用效率；研究确定岸线资源有偿使用标准，发挥岸线资源效益。做好港区供水工程、疏港铁路的规划和引资工作。

突出项目带动。抓好一批基础性项目和临港产业项目建设，以项目建设推动港口经济发展。加大工作力度，推进疏港大道、亚太通用码头建设。争取上级支持，尽快动工建设潮州港公共航道。增加资金投入，完成小红山货运码头建设并投入使用。加强协调服务，确保大唐电厂3、4号机组及其送电工程顺利建成投产，促进风能发电、华丰造气厂二期工程等项目建设。

加强招商引资。建立市场运作机制，引进多元投融资主体，多方筹措资金，加大港口开发建设投入。抢抓国际产业转移加快的有利时机，加强港区特别是重化园区招商引资工作。继续做好跟踪洽谈，争取中海油天然气接收站、潮州港经济区物流中心、华美油脂等一批项目落户港区。

（四）发挥资源优势，做大做强文化旅游产业

加大宣传推介力度。抓住广济桥和牌坊街等文物景点相继开放的有利时机，全方位、多渠道加强宣传推介，全面提升我市文化旅游的核心竞争力和影响力。高标准、有特色办好省第二届粤东侨博会暨潮州市第三届文化旅游节等活动，组团参加第十五届国际潮团联谊年会，以盛事活动为契机，大力宣传推介潮州。深化旅游区域合作，着力打造特色文化旅游品牌，加快把我市建设成为令人向往的国内文化旅游首选目的地。

整合文化旅游资源。争取古城文化旅游区建设纳入上级资助计划。全面完成牌坊街修复工作并对外开放。加快文化特色专馆、华侨博物馆的建设和己略黄公祠、笔架山宋窑遗址等文物景点的修复。整合古城区文化旅游资源，推行旅游优惠套票，不断增强潮州文化旅游吸引力。

推进改革与发展。贯彻实施省委省政府《关于加快旅游业改革与发展建设旅游强省的决定》，促进旅游事业全面发展。加强景区景点建设，提升质量等级，积极发展休闲旅游，争创2个国家4A级景区。引导投资旅游产业，做好星级酒店建设的招商引资，鼓励开发民居客栈，建设旅游咨询服务中心，完善旅游配套设施，解决游客住宿、停车、交通信息导入难等问题。加快发展旅游配套服务业，延伸旅游服务消费。重视旅游服务队伍建设和行业管理，营造良好的旅游发展环境。

推动文化与经济融合。培育具有潮州特色的文化产业集群，促进传统工艺品产业化发展，努力打造广东省（潮州）旅游商品基地，进一步提高旅游商品市场发展水平。不断壮大动漫、包装印刷、光盘音像出版等产业，带动相关配套产业发展。

（五）拓展内外市场，大力发展商贸服务业

努力保持外贸增长。认真落实扶持外贸发展政策措施，积极做好出口退税工作。引导企业提高产品质量档次，促进更多企业获得出口质量许可等各类认证。支持品牌产品和优势产品出口，扩大高新技术产品出口比重。抓住当前国际市场环境深刻变化的契机，引导企业深度开发欧美等传统市场，大力拓展非洲、中东、拉美和俄罗斯等新兴市场。鼓励企业到境外创办营销网点或生产基地，积极发展直接贸易。抓住潮州港被列为对台直航港口的有利条件，促进对台经贸合作。争取设立出口加工区、物流保税区和对台小额贸易市场，鼓励有条件的企业设立保税仓。继续整顿和规范外贸出口秩序，营造公平有序的出口环境。加强口岸建设，优化通关条件。

积极扩大利用外资。借助承办省第二届粤东侨博会等活动，全方位开展港口招商、园区招商、产业招商，把资源优势转化为招商引资优势。重视把引进外资与优化产业结构、提升产业素质结合起来，争取在引进大企业、大项目上实现新突破。加强项目储备、包装和推介，主动承接国际金融危机引发的产业转移和技术转移。抓好在谈项目的跟踪和已批项目的服务，鼓励外资企业增资扩产，不断扩大招商引资规模。

大力开拓国内市场。抓住国家扩大内需的机遇，联合省经贸委在有影响力的大城市举办产品博览会暨经贸洽谈会，积极组织企业参加各类经贸洽谈活动。创新开拓市场的思路和方法，举办拓展内销市场经验交流会和营销讲座，鼓励支持企业建立营销网络和物流体系，全方位开拓国内市场。加快发展生产性服务业，推进特色产业配套市场建设，筹建轻工综合市场、潮汕火车站物流中心。促进生活性服务业发展，加强商业网点和公共服务平台建设，继续实施“万村千乡市场工程”，积极发展中介咨询业和社区服务业。

二、加强投资与环境工作，增强可持续发展能力

实现科学发展、平稳较快发展和可持续发展，必须加大固定资产投资力度，加强生态保护和城市环境建设。

（一）抢抓发展机遇，加快推进重点项目建设

加强重点项目建设基础工作。抓住国家加大投资力度的有利时机，积极扩大固定资产投资，着力抓好关系全局和未来发展的重点项目建设，努力改善发展环境、增强发展后劲。对列入年度新开工计划、投资总额56.4亿元的19个重点项目，抓紧做好各项准备工作，争取尽早开工建设；对列入上级重点建设计划的项目，积极跟进、主动衔接，争取得到上级更多的支持；对列入2009年至2010年储备申报的104个项目，加紧做好规划论证等前期工作。强化项目建设工作责任，完善项目建设协调机制，积极解决项目报批、用地、资金等方面的问题。重视做好重点项目的储备工作，进一步充实重大项目库。

全力加快重点项目建设进度。加紧推进列入年度计划的53个重点项目建设。积极配合做好厦深铁路、汕梅高速公路潮州段建设，抓好省道334线饶平上善至新塘路段大修工程，不断完善交通环境。主动配合推进500千伏、220千伏等输变电工程和2009年配网工程建设，努力提高能源保障水平。加快“三大涝区”整治、韩江南北堤南堤段达标加固等水利工程建设，增强城乡防灾减灾能力。推进市区有线数字电视服务平台、市职业技术学校、市文化艺术馆、市博物馆等一批重点项目建设，进一步改善经济社会发展环境。

（二）突出工作重点，推进生态文明建设

加强环境生态保护。以保护饮用水源水质为重点，加大韩江潮州段、黄冈河和枫江的环境综合治理力度。严格建设项目环保准入制度，控制主要污染物新增排放量。加快市环境监控中心建设，建立完善污染源信息动态管理系统，健全重大环境事件应急监控和预警体系。加快污水处理设施建设，确保桥东污水处理厂和潮安、饶平县城污水处理厂年底前建成投入使用。加强海洋、湿地、林地和地质环境保护，推进生态公益林和沿海防护林等生态工程建设。

抓好节能减排工作。鼓励发展节能产业和生产，推进以陶瓷窑炉为主的节能降耗技术改造，加快淘汰高耗能、低效益的落后生产能力。推行排污许可证制度，对重点环境问题和重点污染企业实行挂牌督办。加大执法力度，严肃查处超标排污等环境违法行为。

促进节约集约用地。落实国土资源管理共同责任制，及时发现和查处违法违规用地行为。完成土地利用总体规划修编，优化城乡用地布局。争取上级用地指标支持，强化规划和年度计划实施管理。加大土地开发整理力度，确保耕地占补平衡。加强旧城区、旧厂房、旧村庄改造，遏制圈地和低水平建设行为，不断提高土地使用效益。

规范矿产资源管理。继续整顿矿产资源开发秩序，落实动态巡查责任制，坚决打击非法开采稀土、瓷土和河砂等行为。完善矿产资源管理制度，规范采矿权审批管理，严格市场准入条件。

（三）加强规划建设管理，全力打造“三宜”城市

重视城市规划工作。尽快完成《潮州市城市总体规划》修编，做好城市重点区域控制性详细规划编制，进一步优化城市发展布局。加快编制《潮州市国家历史文化名城保护规划》，加强古城区保护利用。认真组织好各类规划的实施，坚决查处违反城乡规划的建设行为。

加强城市基础建设。加快外环路、银槐北路、绿榕西路、市开发区工业大道等城市道路建设。推进潮州大桥、韩江东西溪大桥建设，搞好市城区东片建设基础工作，促进城市向东扩展。积极创建国家环保模范城市，巩固发展国家园林城市创建成果。加强城市园林景观建设，改造建设潮州植物园。加快市区排涝渠系改造，力争今年解决内涝问题。推进市城区西片排污管网和垃圾填埋场二期工程等建设，增强城市污染防治能力。

提高城市管理水平。强化道路交通组织管理，完善公交线路和站点设置，做好市区公共汽车运营过渡工作，积极发展城市公共交通。加强对营运市场的监管，打击非法出租车和电动三轮车的经营行为。理顺城市管理体制，整合城管执法队伍，提高执法管理水平。整治城乡结合部环境，治理占道经营和沿街摆卖行为。抓好道路保洁等工作，提高城市美化、净化、绿化和亮化水平。进一步完善各项管理制度，建立健全城市管理长效机制。

三、发展各项社会事业，全面履行政府职责

社会和谐稳定、民生得到保障，是群众的愿望、政府的职责。要进一步采取有力措施，积极构建和谐社会。

（一）提高保障水平，不断促进民生改善

实施积极就业政策。完善城乡劳动就业制度体系、服务体系和多层次职业培训体系。注重城乡统筹就业，认真落实农村劳动力转移政策。加强职业技能培训，拓宽就业渠道，千方百计扩增就业岗位,力争城镇新增就业2.5万人，培训农村劳动力2万人，转移就业4万人。

做好社会保障工作。积极挖掘扩面征缴潜力，加强社保基金管理，确保基金收支平衡。阶段性降低部分社保费率，提高养老保险待遇。推进城镇居民基本医疗保险，争取达到全覆盖目标。规范劳动管理，及时解决处理劳资纠纷，努力构建和谐劳动关系。

改善住房饮水条件。加快经济适用房和廉租房建设，争取一批解困房动工建设和投入使用。认真解决农村群众饮水难和饮水不安全问题，确保农村饮水安全工程和解困工程全部开工建设。加快竹竿山水厂扩建并扩大供水范围，更新改造部分供水管道，改善供水条件，提高供水质量。

完善社会救助体系。继续做好解困工作，扩大社会救助范围和覆盖面。大力发展社会慈善事业，拓宽社会互助渠道。推进社会救助设施和配套设施建设，完善市级救灾物资储备中心。搞好五保户供养和敬老院建设。继续做好对口支援汶川地震灾区恢复重建工作。

（二）发展公共事业，积极建设和谐社会

推进精神文明建设。加强社会主义核心价值体系建设，弘扬新时期潮州人精神。全面开展各类文明创建活动，推进“六好”平安和谐社区等建设，认真做好迎接省文明城市复检工作，继续办好“书香潮州”全民读书节。

发展文化体育事业。积极开展各种形式的文化活动，丰富城乡群众文化生活。加强农家书屋、乡镇文化站建设，完善公共文化服务体系。做好非物质文化遗产保护和利用。抓好各级全民健身场所建设，推动全民健身活动深入开展。办好市第十届体育节、第十四届市运会。

促进教育均衡发展。落实以县为主的管理体制，推动县域义务教育均衡发展。加快义务教育规范化学校建设。积极发展普通高中和职业技术教育，新建扩建10所普通高中学校、5所职业技术学校，确保高中阶段教育普及率70%以上。努力扩大职业技术教育办学规模，不断提高办学质量和水平。推进义务教育教师绩效工资改革，解决代课教师和中小学教师工资福利待遇问题。

提高医疗卫生水平。完善公共卫生服务体系，做好重大疾病防控工作。推进市中医医院、市精神病院、市妇幼保健院新院，以及中心卫生院为重点的镇级卫生院建设，整合建设社区卫生服务中心。加快乡镇卫生管理体制改革。加强妇幼保健和爱国卫生工作。巩固扩大农村合作医疗覆盖面，进一步提高补偿标准。

抓好人口计生工作。狠抓生育节育政策落实，千方百计稳定低生育水平，巩固提高潮安县、饶平县转类升级成果，力争人口计生工作进入全省先进行列。

积极建设平安潮州。坚持打防结合，严厉打击各类严重刑事犯罪和多发性犯罪，大力整治社会治安突出问题。加强和改进信访工作，引导群众按照法定程序合理表达利益诉求，积极防范和妥善处置群体性事件。严格安全生产监督管理，防范重特大安全事故发生。加强基层应急管理工作，积极预防和及时处置突发事件。

统筹发展各项事业。继续做好人才、民族宗教、广播电视、新闻出版、外事侨务、对台、人防、气象、防震、统计、物价、档案、地方志、老龄、残疾人、妇女儿童、国防、民兵预备役等工作，促进各项事业全面发展。

（三）加强政府建设，努力提升管理服务水平

解放思想推动创新。深入开展学习实践科学发展观活动，继续解放思想，坚持改革开放。树立世界眼光，增强危机意识，形成战略思维，坚定发展信心，把贯彻上级精神与发挥我市优势结合起来，用创新的思路和举措，破除制约科学发展的障碍，在应对挑战中不断推动经济社会发展上新水平。

转变作风落实责任。扎实开展“落实推进年”活动，把思想和精力放在思落实、抓落实和促落实上。建立健全服务机制，规范审批程序，简化审批环节，加快办事节奏。加强各级行政服务中心建设，推进网上审批，完善“一站式”服务。强化工作责任制，将保增长、保民生的责任层层分解落实，做到有目标、有标准、有部署、有监督、有检查，确保各项工作落到实处。

深化改革增添活力。推动政府职能转变，理顺部门职责关系。稳步推进行政机构改革和事业单位分类改革，深化综合行政执法改革。搞好药品、医疗服务价格改革。实行用水、用电阶梯式价格，提高水资源费、排污费征收标准。坚持依法治税理财，积极培育税源，完善收入异动预警机制，保持财政收入稳定增长。完善公共财政体制，优化财政支出结构，严格控制一般性财政支出，保障重点项目、民生工程的支出进度和效果。

依法行政勤政为民。自觉接受市人大的工作监督、法律监督和市政协的民主监督，认真办理人大代表议案、建议和政协委员提案。健全行政决策程序，广泛听取各民主党派、工商联、无党派人士意见，发挥工会、共青团和妇联等人民团体作用。抓紧建立依法行政各项配套制度，继续完善社情民意反映制度、听证制度和专家咨询制度。按照“为民、务实、清廉”的要求，加强公务员队伍建设，严格执行廉洁从政各项规定，加快构建开放、动态、创新的惩治和预防腐败体系。深入开展调查研究，积极为民办实事、办好事、解难题，大力弘扬清正廉明、奋发有为的作风。

各位代表！新的一年，是充满挑战的一年，也是蕴含机遇的一年。让我们在省委、省政府和市委的领导下，全面贯彻落实党的十七大精神，深入学习实践科学发展观，解放思想、坚定信心，迎难而上、扎实工作，努力开创潮州科学发展新局面，以优异的成绩迎接新中国成立60周年！
